# 

**Published:** 1841-07-01

**Authors:** 


### MEDICO-CHIRURGICAL REVIEW,

AND

### JOURNAL

OF

### PRACTICAL MEDICINE.

*(NEW SERIES.)*

VOLUME THIRTY-FIVE,

[1st of APRIL to 30th of SEPTEMBER,]

1841.

VOL. XV. of DECENNIAL SERIES.

EDITED

BY JAMES JOHNSON, M.D.

PHYSICIAN EXTRAORDINARY TO THE LATE KING,

AND

HENRY JAMES JOHNSON, ESQ.

LECTURER ON ANATOMY AT THE SCHOOL OF ST. GEORGE'S HOSPITAL IN KINNERTON STREET.

Surgeon General's Office LIBRARY 57475

LONDON:

PUBLISHED BY S. HIGHLEY, 32, FLEET STREET,

*Re-printed in New York, by Mr. Wood.*

1841.

WI
ME 75T

PRINTED BY F. HAYDEN,

Little College Street, Westminster.

### INDEX.

A.

Abdominal inflammations.......... 277
Abscess in the œsophagus.......... 16
Abscess between rectum and vagina.. 24
Abscess in the pelvis.............. 122
Absorption, ulcerative, of cranium .. 5
Absorption of metals into the blood.. 255
Abuses in madhouses............... 151
Action of the poison of fever ...... 50
Acton on the venereal disease ...... 26
Adams on compression of the heart.. 287
Adams on amaurosis............... 479
Administration of iodine .......... 510
Albuminous nephritis ............. 454
Albuminous nephritis, forms of .... 454
Albuminous nephritis, symptoms of, 456
Albuminous nephritis, chronic...... 458
Albuminous nephritis, causes of .... 461
Albuminous nephritis, diagnosis of.. 462
Albuminous nephritis, treatment of.. 464
Albuminous nephritis, complications, 469
Albuminuria ..................... 505
Algiers, French army in............ 213
Alston moor, medical topography of, 289
Alston moor, diseases of ........... 290
Alum, on the use of .............. 222
Amaurosis, temporary ............ 7
Amaurosis, temporary ............ 272
Amaurosis, Adams on ............ 479
Amenorrhœa, electricity in........ 245
America, Combe's notes on ........ 68
America, Combe on ............... 353
Americans, Notes on the .......... 353
Amputation of the lower jaw........ 120
Amputation at the shoulder-joint.... 268
Amputations in the French army.... 213
Analogy between hysteria and gout.. 105
Analysis of the blood, Mandl's........ 178
Anatomy of the arteries, Quain's.... 31
Anatomy & physiology, Cyclopædia of 165
Anatomy of light-giving organs...... 167
Anatomy, application of microscope to 220
Anatomy of the spinal system, Dr. Hall 309
Anchylosis, rapid extension for ..... 214
Andral's humoral pathology........ 177
Andral on humorism............... 500
Andral on the blood in hæmorrhages, 505
Andral on the buffy coat .......... 506
Andral on fever.................. 559
Aneurism ...................... 18
Aneurism, Mr. Porter on .......... 388
Aneurism, causes of .............. 391
Aneurism, internal................ 393
Aneurism, thoracic................ 394
Aneurism of abdominal aorta ...... 395
Aneurism, treatment of............ 396
Aneurism, on the use of pressure in.. 399
Aneurism, treatment of, by ligature.. 401
Aneurism, diffused................ 403
Aneurism, traumatic.............. 405
Aneurism, failure of operation for... 406
Aneurismal varyx ................ 415
Animal luminousness.............. 165
Animal light, properties of. ........ 165
Animal luminousness, theories of.... 168
Animal luminousness, uses of ...... 168
Animal magnetism, Majendie on .... 539
Ano, fistula in.................... 269
Anterior chamber, piece of steel in the 131
Antimony, tart. of, in hæmoptysis .. 190
Aorta, aneurism of the ............ 18
Aorta, double tic-tac sound in the .. 189
Apparatus for fractured clavicle .... 547
Application of the microscope to anatomy ........................ 220
Arsenic, on poisoning by .......... 221
Arsenic in bones, Rees on.......... 255
Arsenical cigarettoes for phthisis.... 519
Arteries, Quain's anatomy of the.... 31
Arthritis, puerperal form of ........ 141
Artificial pupil, operation for ...... 128
Ascarides, treatment of............ 526
Ashwell on enlargement of the female breast ........................ 260
Asphyxia, congenital, Dr. Hall on... 319
Asphyxia, secondary, Dr. Hall on ... 320
Aura epileptica .................. 226
Auscultation, Raciborski on........ 183
Auscultatory test for iron.......... 515
Azores, Buller's Winter in the...... 93

B.

Babington on epilepsy ............ 224
Bagneres de Bigorre ............96 484
Banner on injuries of the skull...... 113
Barèges ........................ 484
Barlow's observations on phthisis ... 263
Basis cranii, fracture of the ........ 7
Baths of Ischia .................. 47
Baths of Furnas.................. 97
Benoit's case of hermaphrodism ..... 537
Bethlem Hospital ...............90 159
Bethlem Hospital, report from...... 561
Bigorre, Bagneres de.............. 96
Biliary calculi, convulsive cough from 21
Biliary concretions, Mallett on...... 125
Biot on diabetes mellitus .......... 203
Bird on the value of electricity...... 239
Birmingham Eye Infirmary, report from the ...................... 128

Birmingham Infirmary, report from.. 125
Blacks, mortality of the............ 551
Bladder, rupture of the ........... 441
Blood, chemical analysis of the...... 178
Blood, deficient in fibrine.......... 181
Blood, specific density of the ....... 183
Blood, absorption of metals into the.. 255
Blood, changes of the, in hæmorrhages 505
Blood, dissolved state of the........ 506
Blood, presence of pus in the ...... 506
Blood, state of the, in fever........ 559
Blood, state of the, in inflammation.. 560
Bodily organs, influence of attent. on, 491
Bombay Hospital, report from the... 564
Bone passed from the urethra...... 259
Bones, Rees on arsenic in.......... 255
Boston, vital statistics of .......... 550
Boston, diseases of................ 553
Brain, ramollissement of the........ 11
Brain, laceration of the............ 113
Brain, diseases of the.............. 172
Brain, Brigham on the ............ 489
Brain, inflammatory diseases of the.. 490
Braithwaite's retrospect of medicine.. 569
Breschet on hydrophobia .......... 198
Brett on the surgical diseases of India 383
Brigham on the brain .............. 489
Brodie on ununited fracture........ 575
Buffy coat, formation of the........ 180
Buffy coat, Andral on the.......... 506
Bullar râle ...................... 186
Buller's Winter in the Azores ...... 93
Bullet, curious course of a ......... 565
Burns, Earle on .................. 575
Bursæ mucosæ, subcutaneous div. of, 574

C.

Cæsarian operation ............. 60, 432
Canton Ophthalmic Hospital........ 275
Cardia, stricture of the ............ 22
Cardia, action of.................. 312
Carriere on gastralgiæ ............ 516
Cartilage, diagnosis of ulceration of.. 141
Cartilage, treatment of ulceration of, 143
Castellamare ..................... 51
Castellamare, mineral waters of..... 52
Cataract, operation for............ 385
Cataract, Dr. Watson on .......... 136
Catheter, the self-guiding ......... 139
Catheter, new kind of ............ 574
Catheterism, Costello on .......... 137
Catoptrical exploration of lens...... 136
Cauterets......................... 485
Cerebral affections of children ...... 253
Cerebral system, Dr. Hall on........ 307
Cerebral congestion .............. 505
Cerebral disease, treatment of ...... 569
Certificate system ................ 31
Chapman on club-foot ............ 145
Character of Velpeau.............. 220
Chemical analysis of the blood...... 178
Chest, hydatids in the ............ 194
Children, cerebral affections of...... 253
Chlorosis, use of iron in .......... 515
Choking, treatment of ............ 312
Chomel on rheumatism............ 523
Chorea ......................... 241
Chowne's medical oration.......... 481
Christison on diabetes............. 281
Chronic pleurisy, Dr. Hope on...... 295
Chronic hydrocephalus, crude mercury in........................ 572
Churchill on operative midwifery.... 53
Chusan ........................ 593
Chusan, description of ........... 593
Chusan, climate of................ 594
Circocele, treatment of, by operation, 212
Clark's influence of climate ........ 93
Clavicle, apparatus for fractured .... 547
Climate, Clark's influence of........ 93
Climate of Nice .................. 486
Clinical remarks on rheumatism .... 523
Clinical observations of Mr. Morgan's, 556
Clinical remark of Velpeau......... 562
Club-foot, memoir on ............. 33
Club-foot, Chapman on............ 145
Cock on structure of the urethra .... 249
Cod-liver oil, internal use of........ 222
Coldstream on animal luminousness.. 165
Colon, spasm of the .............. 23
Combe on moral philosophy...... 68 353
Combe's notes on North America.. 68 353
Complex labours.................. 237
Compression of the heart.......... 287
Consultations.................... 558
Cooper, Sir A., sketch of .......... 80
Cooper, Sir A., the late ........... 265
Cooper's, Sir A., early cases........ 558
Copaiba and turpentine, pills of .... 222
Cord, ligature of the .............. 64
Cord, decadence of the ............ 64
Cornea, sloughing of the........... 385
Cornea, Mr. Jones on the.......... 146
Corneæ, staphyloma .............. 146
Cornea-sclerotic junction, tumor at, 130
Corpus luteum .................. 422
Costello's Cyclopædia ............ 135
Costello on catheterism............ 137
Costello's self-guiding catheter...... 139
Cough from biliary calculi ......... 21
Cowan's register for hospitals ...... 132
Cowper's gland, disease of.......... 30
Cox on Naples ................... 44
Crampton on spina bifida .......... 288
Craniotomy...................... 59
Cranium, ulcerative absorption of the, 5
Cranium, necrosis of the .......... 6
Cranium, fracture of the .......... 548
Crowther on mad-houses .......... 155
Cruveilhier on the sounds of the heart, 564
Cure of spina bifida ............... 288
Cure for amaurosis ............... 479

Curious course of a bullet.......... 565
Curvature of the spine, Guerin on .. 33
Curvature of the spine, memoir on .. 33
Curvature of the spine, Tuson on ... 33
Curvature of the spine, mechanical treatment of.................... 38
Cyclopædia for practical surgery .... 135
Cyclopædia of anatomy & physiology, 165
Cynanche, guaiacum for ........... 570
Cynic spasm, Dr. Hall on.......... 316

D.

Dangers of paracentesis thoracis .... 193
Dead organic matter, fever poison from 35
Death of homœopathy ............. 488
Deficiency of the pectoral muscles ... 260
Deficient development of parts...... 8
Dendy's philosophy of mystery...... 65
Delirium tremens, dangers of opium in 573
Dental surgery, Mr. Waite on ...... 173
Development, deficient from injury .. 8
Diabetes mellitus, M. Biot on ...... 203
Diabetes, Christison on............. 281
Diabetes, determination of.......... 283
Diabetes, rapidly fatal ............. 284
Diarrhœa, burnt rhubarb for........ 572
Dieffenbach on stammering ........ 206
Diffused aneurism ................ 403
Diplopia, Jones on................ 148
Disease of Cowper's gland.......... 30
Diseases of women, Dr. Laycock on, 100
Diseases, natural order of.......... 150
Diseases of India, Mr. Brett on the .. 383
Diseases of the kidneys, Rayer on.... 452
Dislocation, partial, of the lens ...... 131
Dislocations, relative frequency of .. 549
Dodd's amputation of the lower-jaw, 120
Dryness of the pia mater .......... 9
Dubois on the signs of pregnancy.... 543

E.

Earle on burns .................... 575
Eastern penitentiary in Pennsylvania, 360
Ecchymosis of the eyelids.......... 114
Edinburgh, fever in................ 268
Edinburgh Dispensary ............. 268
Edinburgh Eye Dispensary ......... 271
Effusion of serum in the head ...... 9
Elater ............................. 165
Elbow, fractures of the ............ 548
Electricity, on the value of ........ 239
Electro-acupuncture for neuralgia ... 202
Emetics, for hooping-cough ........ 523
Enlargement of the female breast.... 260
Enlargements of the spleen ........ 197
Epidemic meningitis .............. 527
Epidemic meningitis, symptoms ..... 528
Epilepsy, observations on .......... 224
Epilepsy, treatment of ........... 227
Epine, memoire sur les deviations de l', 33
Epithelium, structure of the........ 447
Epithelium, component parts of the.. 449
Epithelium lining the mouth........ 450
Epps on the vaccine virus.......... 285
Eradication of inverted eyelashes .... 271
Erysipelas, Velpeau on ............ 508
Erysipelas, new application to ...... 508
Erysipelas, remedies for ........... 509
Escape of the vitreous humour...... 130
Etiology of club-foot .............. 33
Europe, Gibson's rambles in......... 80
European constitution in India...... 383
Ewart on the Alston Moor.......... 289
Expiratory murmur ................ 185
Extension, treatment of anchylosis by 214
Eye, case in which one was very large, 129
Eye Dispensary of Edinburgh ....... 271
Eyelashes, inverted, eradication of .. 271
Eyelids, ecchymosis of the.......... 114

F.

False membrane, nature of ........ 560
Farr's mortality of lunatics ........ 82
Farr's medical guide to Nice........ 93
Fatal wound of the vertebral artery . 209
Fatal cases in midwifery ........... 239
Fauces, irritation of, for vomiting.... 314
Female breast, enlargement of the .. 260
Females, morbid appearances in ..... 561
Femur, fracture of the ............ 545
Fever poison from dead organic matter 35
Fever poison, sources of... ......... 48
Fever poison, action of the ........ 50
Fever, pathology of .. ............. 153
Fever in Edinburgh ............... 268
Fever, M. Andral on .............. 559
Fever, symptomatic, blood in ....... 559
Fevers of Boston ................. 554
Fevers, eruptive.................. 554
Finger, re-union of a............... 281
Fistula in ano.. .................. 269
Fistula lachrymalis, treatment of .... 566
Fistulæ, recto-vaginal, treatment of.. 210
Flooding labours.................. 239
Florence, hospitals of.............. 487
Forceps, use of the... ............. 56
Fore-arm, fracture of the .......... 548
Forget on modern humorism........ 497
Forget's letter to M. Andral ........ 497
Forget on incurable diseases........ 530
Fracture of the basis cranii ........ 7
Fracture of the femur ............. 545
Fracture of the leg................. 546
Fractures, relative frequency of...... 549
France, progress of medicine in ..... 216
France, Lee's memoranda on ....... 482
France, vaccination committee of.. .. 540
French army, amputations in the.... 213
French and English surgery ........ 483
French watering-places ............ 483
French academy, scene in the ...... 542
Fungoid tumor in the pharynx...... 15

Funis umbilicalis.................. 61
Funis, growth, &c. of the ........ 61
Funis, knots of the .............. 62
Funis, coiling of the ............. 63
Furnas, baths of.................. 97
Furnas, climate of................ 97

G.

Gall-bladder, obstruction of the .... 20
Gall-bladder, permanent spasm of .. 20
Ganglionic system, Dr. Hall on ..... 317
Gangrena senilis, case of .......... 221
Gastralgiæ, M. Carriere on ........ 516
Gastrorrhea........................ 379
Germany, Lee's memoranda on....... 482
Gibson's rambles in Europe ........ 80
Glanders in the human subject...... 279
Gonorrhœa, virulent ............... 26
Gonorrhœa, speedy suppression of .. 28
Gonorrhœa, diagnosis of .......... 29
Gonorrhœa, specific for............ 576
Gravid uterus...................... 424
Griffin on abdominal inflammation .. 277
Guaiacum for cynanche............. 570
Guerin on curvature of the spine.... 33
Guerin on rachitis................. 285
Guernsey, Redstone's guide to...... 164
Guernsey, climate of .............. 164
Guide to Guernsey................. 164
Guthrie, Mr. sketch of............. 81
Guy's hospital reports ............ 225
Guy's hospital lying-in charity...... 233
Guy's, the Nockemoff's of.......... 556

H.

Hæmoptysis, tart. antim. in........ 190
Hæmorrhage, Porter on............. 388
Hæmorrhage, its treatment ........ 390
Hæmorrhage, secondary ........... 408
Hæmorrhage, accidental .......... 439
Hæmorrhage, secondary ........... 115
Hæmorrhage after operations ...... 268
Hæmorrhage, nitrate of silver for.... 575
Hæmorrhages, changes of the blood in 505
Hæmorrhagic diathesis ............ 276
Hæmorrhoidal tumors, Dr. Hall on.. 315
Hall, Dr. Marshall, on the nerv. system 305
Hall, Dr. on anatomy of the spinal system........................ 309
Hall, Dr. on congenital asphyxia.... 319
Hanwell, report from............... 84
Hanwell hospital .................. 160
Haslar hospital ................... 158
Head, chronic pain in the.......... 4
Head, effusion of serum within the.. 9
Head, injuries of the ............. 113
Head, injuries of the, Mr. Sharp on.. 344
Heart, compression of the......... 287
Heart, Cruveilhier on the sounds of.. 564
Hermaphrodism, case of........... 537
History of Sir A. Cooper .......... 265
History of St. Vitus .............. 490
Homœopathy, death of ............ 488
Hooping-cough, Trousseau on...... 522
Hooping-cough, cure of .......... 523
Hope on chronic pleurisy .......... 295
Hospitals, Cowan's register for...... 132
Hospitals, construction & management 338
Hospitals of Florence.............. 487
Howship on surgical diseases ....... 1
Hughes on the cerebral affections of children ...................... 253
Human subject, glanders in the ..... 279
Humerus, fracture of the .......... 547
Humoral pathology, M. Andral's.... 177
Humorism, modern, Forget on...... 497
Hunter on inverted eyelashes ....... 271
Hunter on temporary amaurosis .... 272
Hydatid tumors of the liver ........ 196
Hydatids in the chest.............. 194
Hydrocephalus, crude mercury in ... 572
Hydrophobia, Breschet on.......... 198
Hysteria, physiology of ........... 102
Hysteria and gout, analogy between.. 105
Hysteria, treatment of ............ 107
Hysteria, predisposing causes of .... 108

I.

Imperforate jejunum .............. 559
Incipient cataract, diagnosis of...... 157
Incised wound of the knee-joint .... 257
Incisions for pain in the head ...... 4
Incurable diseases, treatment of .... 530
India, Brett on the diseases of ...... 383
India, European constitution in ..... 383
Indian operation for stone ......... 387
Induction of premature labour...... 53
Inflamed testis, compression of a .... 29
Inflammation of the thyroid gland .. 13
Inflammation of the veins.......... 19
Inflammation of the liver .......... 20
Inflammation, pathology of ........ 153
Inflammation, Andral on .......... 559
Inflammation, state of the blood in .. 560
Influence of climate .............. 93
Influence of attention on bodily organs 491
Injury by lightning................ 10
Injuries of the skull, Banner on ..... 113
Insane poor, treatment of the ...... 370
Insanity, notes on ................ 353
Insanity, is it a fatal disease? ...... 85
Insanity, probable duration of...... 89
Insanity, probability of recovery from, 89
Insanity, prevalence of ........... 111
Insanity, pathology of ............ 561
Instrumental labour .............. 57
Intermittent fevers, narcotine in .... 564
Intermittent fevers, piperine in...... 570
Internal use of cod-liver oil ........ 222
Intraction of a bougie ............ 314
Intussusception, cure of .......... 24
Iodine, administration of .......... 510

Iodine, where useful ............... 511
Iodine, salivation from ............. 571
Iron, auscultatory test for.......... 515
Ischia, baths of .................... 47
Italy, Lee's memoranda on ......... 482
Itch, bichloride of mercury for.. ... 566
Ischia .... ........ ............ 49
Ivory, formation of the............. 445

J.

Jacobs on spontaneous combustion .. 529
Jaw, amputation of the lower ...... 120
Jejunum, imperforate .............. 559
Joints, moveable bodies in.......... 116
Jones on the cornea ............... 146
Jones on diplopia ................. 148

K.

Kidneys, Rayer on diseases of the.... 452
Kidneys, granulated texture of the .. 455
Knee-joint, incised wounds of the.... 257
Knee-joint, Elston on wounds of the.. 257
Knee-joint, disease of the .......... 269
Koecker, Dr. ...................... 600

L.

Labour, lingering .................. 430
Labour, instrumental............... 434
Labour, premature, induction of .... 436
Labour, complex.................... 437
Labour, premature. ............... 53
Labour, instrumental............... 57
Lacteal system, Lane on the ........ 169
Largeness of one eye .............. 129
Larynx, suppuration in the ... .... 13
Lawrence, Mr. sketch of .......... 80
Laycock on the diseases of women ... 100
Lee's memoranda on France, &c. .... 482
Leg, fractures of the .............. 546
Lens, ossification of the............ 129
Lens, partial dislocation of the...... 131
Lens, catoptrical exploration of the.. 136
Lever, use of the ................. 58
Lever's report ..................... 233
Ligature of the cord ... .......... 64
Light-giving organs, anatomy of .... 167
Lightning, injury by ............... 10
Liston, description of Mr........... 21
Lithotomy in Naples ............... 212
Liver, Thomson on diseases of the ... 163
Liver, diseases of the .............. 171
Liver, hydatid tumors of the ...... 196
Lodgings for students.. ........... 174
Lumbrici........................... 586
Lunatics, Farr on the mortality of .. 82
Lunatics, admission and discharge of, 91
Lyons' statistics of rheumatism ..... 112
Lymphatic system, Lane on the ..... 169

M.

Macartney on moveable bodies in joints 116
Mackenzie on strabismus .......... 493
Madhouses, Dr. Crowther on ....... 155
Madhouses, abuses in............... 156
Magendie's treatment of neuralgia ... 202
Magendie on animal magnetism ..... 539
Malignant disease of the œsophagus.. 15
Mallett on biliary concretions ...... 125
Marseilles, hospitals at ............ 483
Massey on fluid in the thyroid body.. 257
Measles in 1839 ................... 126
Medical jurisprudence, notes on ..... 354
Medical guide to Nice............... 93
Medical topography of Shrewsbury .. 110
Medical pathology and therapeutics .. 218
Medical Society, oration to the ..... 481
Medical topography of Alston Moor.. 289
Medicine, progress of, in France .... 216
Medicine, retrospect of ............ 569
Medico-Chir. Transactions, Provincial 109
Memoire sur le rachitism .......... 33
Memoire sur le torticollis ancien ... 33
Memoire sur les deviations de l'epine, 33
Memoire sur les piedbots congenitaux, 33
Memoranda on France, Italy, and Germany........................ 483
Meningitis, epidemic .............. 527
Menstruation, Raciborski on........ 538
Midwifery, Dr. Ramsbotham on .... 418
Membrana decidua....... ........ 424
Metallic tinkling sound ............. 188
Metals, absorption of, into the blood, 255
Metastatic abscesses, cause of ...... 562
Metastatic abscesses, result of ...... 563
Metastatic abscesses, treatment of ... 563
Microscope, application of, to anatomy 220
Microscopic observations on milk.... 205
Middlemore's report ............... 128
Midwifery, Dr. Churchill on........ 52
Milk, microscopic observations on .. 205
Mineral waters of Naples .......... 44
Mineral Waters ................... 48
Modern humoral pathology ........ 177
Modern humorism, Forget on ...... 497
Monson's report from Bethlem...... 561
Moral therapeutics................. 511
Moral prophylaxy, Combe on ....... 68
Moral philosophy .... ............ 68
Moral philosophy, Combe on ....... 353
Morbid appearances in females...... 561
Morgan's clinical observations ...... 556
Morgan on fistula lachrymalis ...... 566
Morphia, use of, in tetanus ........ 224
Mortality of lunatics ............... 82
Mortality of the blacks ............. 551
Mortality of different sexes ........ 552
Mortality of different seasons ....... 553
Moveable bodies in joints .......... 116
Mucous membranes, use of alum in.. 222
Musk in pneumonia ............... 193
Mystery, philosophy of ............ 65

N.
Naples, Dr. Cox on ............... 44
Naples, temperature of ............. 44
Naples as a Winter residence......... 46
Naples, diseases benefited by......... 46
Naples, lithotomy in ............... 212
Narcotine in intermittent fevers .... 564
Nasmyth on the teeth ............. 442
Nature of false membrane........... 560
Nature of presentations............. 238
Navy, mortality in the ............. 170
Neapolitan hospitals, med. treat. in.. 488
Necrosis of the cranium.............. 6
Nervous system, diseases of the...... 555
Nervous system, Dr. Hall on ....... 305
Neuralgia, Magendie's treatment of .. 202
New covering for pills ............. 222
New hydraulic machine.......... .. 600
Nice, medical guide to ............. 93
Nice, climate of.................93, 486
Nice, diseases benefited by ......... 94
Nitrate of potass in rheumatism .... 523
Nitrate of silver for hæmorrhage .... 575
Nockemoff's of Guy's............... 556
Norris's hospital report ............. 445
Notes on North America, by Combe.. 353
Notes on the Americans............. 353
Notes on insanity ... ..... ...... 353

O.
Obstetric medicine, Ramsbotham on, 418
Observations, microscopic, on milk .. 205
Observations on epilepsy ... .... . 225
Observations on phthisis ........... 263
Odontography, Mr. Owen on ....... 442
Œsophagus, stricture of the ........ 14
Œsophagus, inflammatory stricture of 14
Œsophagus, malignant disease of.... 15
Œsophagus, abscess in the ......... 16
Old people, pneumonia in ........... 532
Operative midwifery, Dr. Churchill on 52
Operation, treatment of circocele by.. 212
Operation for cataract ............. 385
Operation for stone in India........ 387
Operation for artificial pupil......... 128
Operation for strabismus ........... 493
Ophthalmic hospital of Canton....... 275
Opium, dangers of, in delirium tremens 573
Optic nerve, temporary affection of.. 7
Oration, Dr. Chowne's ............. 481
Organs of respiration, diseases of the, 555
Organs of circulation, diseases of the, 555
Orifices, guards of ................. 311
Ossification of the lens ............. 129
Owen on odontography.. .......... 442

P.
Pain in the head, chronic .......... 4
Paracentesis thoracis, dangers of .... 193
Paracentesis thoracis, Sedillot on .... 535
Paralysis ......................... 242
Paralysis, rheumatic ............ .. 243
Paralysis from affection of the brain.. 243
Paralysis from local injury........... 244
Paralysis, excision of tendon for .... 574
Paris, medical students in........... 174
Paris, climate of.................... 482
Parisian hospitals .................. 174
Parisian dissecting rooms ........... 176
Parts, deficient development of...... 8
Parturition ........................ 428
Parturition, classification of ........ 429
Pathological investigations, results of, 489
Pathology of fever .................. 153
Pathology of inflammation .......... 153
Pathology of true spinal system ..... 315
Pathology of insanity............... 561
Pau, description .................... 99
Pau, climate of .................... 99
Pau, healthiness of................. 100
Pectoral muscles, deficiency of the .. 260
Pelvis, description of the ........... 420
Pelvis, deformed.................... 420
Pelvis, case of abscess in the........ 122
Pennsylvania, penitentiary in ...... 360
Pennsylvania Hospital, report from.. 545
Perforation of the skull ............. 114
Pericardium, paracentesis of the .... 535
Peritonitis simulating psoas abscess .. 25
Perspirations, profuse, tannin for.... 571
Perth Infirmary .................... 268
Pertussis, Trousseau on............. 522
Pharynx, fungoid tumor in the...... 15
Philosophy of mystery ............. 65
Phrenology and education.......... 374
Phthisis, treatment of ............. 519
Phthisis, arsenical cigarrettos for .... 519
Phthisis, various remedies for ...... 520
Phthisis, venesection in.............. 521
Phthisis, use of iodine in .......... 521
Phthisis, Barlow on . ............ .. 263
Phthisis, influence of climate on .... 263
Phthisis, influence of employment on, 263
Phthisis, pathological observations on, 264
Phthisis, practical deductions ...... 265
Physical education..... ........... 373
Physiology of hyteria............... 102
Physiology of the spinal system ..... 310
Pia mater, dryness of the .......... 9
Piles, pitch for .................... 572
Pills, new covering for ............. 222
Pills of copaiba .................... 222
Piorry on the double tic-tac sound in the aorta...................... 189
Piperine in intermittents .......... 570
Pitch for piles...................... 572
Plague in a man-of-war, not contagious 596
Plague, cases of ................... 597
Pleurisy, chronic, Dr. Hope on...... 295
Pleurisy, chronic, symptoms of...... 295
Pleurisy, chronic, terminations of.... 296
Pleurisy, diaphragmatic............. 301

Pneumonia, musk in .............. 193
Pneumonia in old persons.......... 532
Poisoning by arsenic ............... 227
Poor, residences of the .......... .. 370
Population, variations of .......... 110
Porter on aneurism................. 389
Potass, nitrate of, in rheumatism.... 523
Practical surgery, cyclopædia of .... 135
Pregnancy, signs of ............... 543
Premature labour, induction of ..... 53
Preternatural labour ............... 237
Prevalence of insanity ............. 111
Progress of medicine in France...... 216
Protracted labours ................. 236
Provincial medico-chirurgical trans... 109
Psoas abscess, peritonitis simulating.. 25
Psychological causes of disease...... 511
Puerperal form of arthritis ......... 141
Puerperal convulsions ...... ...... 238
Punctured artery, how it heals...... 389
Pus, presence of, in the blood ...... 506
Pus, nature of..................... 560
Pylorus, scirrhus of the............ 18
Pyrosis idiopathica, Dr. West on .... 376
Pyrosis, nature of ................ 377
Pyrosis, post-mortem appearances .. 380
Pyrosis, treatment of.............. 381

Q.

Quain's anatomy of the arteries .... 31

R.

Rabies, transmission of ............ 199
Rachitis, memoir on ............... 33
Rachitis, Dr. Guerin on............ 285
Raciborski on auscultation ........ 183
Raciborski on menstruation ........ 538
Rambles in Europe................. 80
Ramollissement of the brain........ 11
Ramsbotham on midwifery ........ 418
Rapid extension for anchylosis ...... 214
Rayer on diseases of the kidneys.... 452
Recto-vaginal fistulæ, treatment of .. 210
Rectum, abscess between it and vagina 24
Rectum, pain of, and remedy ...... 314
Redstone's Guernsey guide ........ 164
Rees on the urine ............... 247
Rees on arsenic in the bones........ 255
Register for hospitals.............. 132
Report from the Western Lying-in Hospital ............. ........ 64
Report from Birmingham Infirmary.. 125
Representative system ............ 279
Retained placenta ................ 238
Retrospect of Medicine ............ 569
Re-union of a finger .............. 281
Reveillé-Parise on moral therapeutics 511
Rheumatic paralysis ........ ..... 243
Rheumatism, statistics of .......... 112
Rheumatism, Chomel on ........ .. 523
Rheumatism, nitrate of potass in.... 524
Rheumatism, acute, treatment of.... 524
Rome, medical practice of... ...... 488
Roux on gangrena senilis .......... 221
Rupture of uterus ................ 238
Rutnagherry, report from.......... 565

S.

St. Luke's Hospital................ 159
St. Vitus, history of .............. 490
Salivation from iodine ............ 571
Sandwich Islanders, skulls of........ 367
Savages, skulls of ................. 366
Scarlatina, second attacks of.. (E. L.) 33
Scene in the French Academy ...... 542
Scurvy........................... 565
Secale cornutum, Wardleworth on the 161
Secondary hæmorrhage............ 408
Sedillot on paracentesis thoracis .... 535
Serum, effusion of, in the head...... 9
Sharp, Mr., on injuries of the head .. 344
Shattuck on the statistics of Boston.. 550
Shoulder joint, amputation at the.... 268
Shrewsbury, medical topography of.. 110
Skull, Banner on injuries of the .... 113
Skull, perforation of the .......... 114
Skull, division of, into regions ...... 489
Sloughing of the cornea .......... 385
Small-pox after vaccination ........ 564
Sounds of the heart, Cruveilhier on.. 564
Soul, Dr. Hall, on the seat of ...... 306
Spasm of the colon................ 23
Spasm of the sphincter ani ........ 23
Spasm, cynic, Dr. Hall on. ........ 316
Specific density of the blood........ 183
Specific for gonorrhœa .............. 576
Speculum, love of the ............ 30
Sphincter ani, spasm of the ........ 23
Spina bifida, cure of .............. 288
Spinal system, pathology of ........ 315
Spleen, enlargements of the........ 197
Spontaneous combustion .......... 529
Stammering, Dieffenbach on........ 206
Stammering, division of the tongue for 206
Staphyloma corneæ ............... 146
Statistical returns, Tulloch's........ 170
Statistics of rheumatism .......... 112
Statistics, vital, of Boston.......... 550
Steam apparatus.................. 576
Steel, a piece of, in the anterior chamber .......................... 131
Stevens' synopsis ................ 150
Stimulating treatment of cerebral disease ......................... 569
Stomach, spasm of the ............ 21
Stomach, purulent effusion in the.... 22
Stomach and bowels, diseases of the.. 173
Stomach, ulceration of the ........ 260
Stomach tube, mode of passing...... 313
Stone, operation for, in India ...... 387
Strabismus, unfavourable results of operation for .................. 495

Strabismus, Mackenzie on.......... 493
Stricture of the œsophagus ........ 14
Stricture of the œsophagus, spasmodic 14
Stricture of the cardia cured ........ 22
Stricture of the urethra, Cock on.... 249
Structure of the epithelium ........ 447
Suppuration in the larynx.......... 13
Surgical diseases of India, Brett on the 383
Surgical diseases, Howship on ...... 1
Sutures in savages................ 367
Symphyseotomy.................. 61
Synopsis, Mr. Stevens' ............ 150
Synovia, want of.................. 559
System of certificates ............ 31

T.

Tænia, treatment of .............. 527
Table of the nervous system—Dr. Hall 307
Tannin for profuse perspirations .... 571
Taylor on poisoning by arsenic...... 227
Taylor on metals in the blood ...... 256
Teeth, Nasmyth on the ............ 442
Teeth, Owen on the .............. 442
Temporary amaurosis.............. 7
Temporary amaurosis ............ 272
Test, auscultatory, for administering iron .......................... 515
Testis, inflamed, compression of a .. 29
Tetanus, use of morphia in ........ 224
Therapeutics, moral .............. 511
Thorax and abdomen, wound of .... 564
Thyroid gland, inflammation of the.. 13
Thomson on diseases of the liver .... 163
Thomson on hæmorrhage after operation ....................... 268
Thyroid body, fluid in the.......... 257
Tibial vein, inflammation of the .... 19
Todd's cyclopædia ................ 165
Torticollis ancien, memoire sur...... 33
Tracheotomy, Trousseau on ........ 191
Transmission of rabies ............ 199
Traumatic aneurism .............. 405
Traumatic tetanus cured .......... 565
Treatment of enlargements of spleen.. 197
Treatment of phthisis ............ 519
Treatment of worms.............. 526
Trousseau on tracheotomy.......... 191
Trousseau on hooping cough........ 522
Tulloch's statistical returns ........ 170
Tumor at the cornea-sclerotic junction 130
Turning ...................... 56
Tuson on curvature of the spine .... 33
Typhus, second attacks of.... (E. L.) 33

U.

Ulceration of cartilage, diagnosis of.. 141
Ulceration of cartilage, treatment of.. 143
Ulceration of the stomach.......... 260
Ulcerative absorption of the cranium 5
Umbilicus, position of the.......... 63
Ununited fracture, Brodie on ...... 575
Urea, on the presence of .......... 282
Urethra, divisions of the .......... 138
Urethra, Cock on stricture of the.... 249
Urethra, bone passed from the...... 259
Urethra, discharges of blood from the 569
Urinary organs, diseases of the...... 556
Urine, Dr. Rees on the ............ 247
Urinous vomiting .......... ..... 273
Use of alum .................... 222
Uterine hæmorrhage, ergot of rye in . 162
Uterus, rupture of................ 238
Uterus, development of the........ 427
Uterus, rupture of the ............ 441

V.

Vaccination committee of France .. 540
Vaccination, small-pox after .. .... 564
Vaccine virus, preservation of . .... 285
Vagina, abscess between rectum and 24
Value of electricity .............. 239
Variations of population .......... 110
Varicose aneurysm................ 415
Various remedies for phthisis ...... 520
Vectis, use of the ................ 58
Veins, inflammation of the ........ 19
Velpeau, character of ............ 220
Velpeau on erysipelas.............. 508
Velpeau's clinical remarks ........ 562
Velpeau's treatment of metastatic abscess ...................... 563
Venereal disease, Acton on the...... 26
Version ........................ 50
Vertebral artery, fatal wound of the.. 209
Vibratory râle.................... 187
Vital statistics of Boston ........ .. 551
Vitreous humour, escape of the .... 130
Volage, nosological report from the.. 593
Vomiting, urinous .............. .. 273
Vomiting, mechanism of .......... 316

W.

Wainwright's case of abscess in pelvis 122
Waite on dental surgery.......... 173
Walking the hospitals ............ 175
Want of synovia ................ 559
Ward's topography of Shrewsbury .. 110
Wardleworth on the secale cornutum 161
Waterbrash, Dr. West on .. ...... 376
Watson on cataract .............. 136
West on pyrosis idiopathica........ 376
Western lying-in hospital .......... 65
Wilmot on the hæmorrhagic diathesis 276
Winter in the Azores ............ 93
Woodward, Dr. portrait of ........ 357
Women, Laycock on the diseases of.. 100
Worcester lunatic hospital.......... 356
Worms, treatment of.............. 526
Wound of the vertebral artery .... 209
Wound of thorax and abdomen .... 564

### BIBLIOGRAPHICAL RECORD.

1. The Anatomy of the Arteries of the Human Body, with its Applications to Pathology and Operative Surgery, in Lithographic Drawings, with Practical Commentaries. By RICHARD QUAIN, Professor, &c. The delineations by JOSEPH MACLISE, Esq. Surgeon. Part VI. With Letter-press. 1841.

2. Guy's Hospital Reports. No. XII. April, 1841. Edited by Drs. BARLOW and BABINGTON. S. Highley, 1841.

3. Elements of Medicine. Vol. II. On Morbid Poisons. By ROBERT WILLIAMS, M.D. Trin. Col. Cambridge, President of the Royal Medical and Chirurgical Society, &c. and Senior Physician to St. Thomas's Hospital. Octavo, pp. 686. Bailliere, 1841.

4. A Practical Treatise on Diseases of the Liver and Biliary Passages. By WILLIAM THOMSON, M.D. One of the Physicians to the Royal Infirmary, &c. Octavo, pp. 306. Maclachlan and Stewart, Edinb. 1841.

5. Edinburgh Monthly Journal of Medical Science. Edited by Jos. ROSE CORMACK, M.D. No. IV. April 1841. Maclachlan and Co. Ed. 1841.

6. The Sources and Mode of Propagation of the Continued Fevers of Great Britain and Ireland. By WILLIAM DAVIDSON, M.D. Senior Physician to the Glasgow Royal Infirmary, &c. Re-printed from the British and Foreign Medical Review. Churchill, London, 1841.

7. A complete Practical Treatise on Venereal Diseases, and their immediate and remote Consequences. Including observations on certain Affections of the Uterus attended with Discharges. By WILLIAM ACTON, late Externe of the Female Venereal Hospital, Paris. Octavo, pp. 410. With a Volume of Plates. Renshaw, Strand. 1841.

8. An Essay on the Chemical, Botanical, and Parturient Properties of the Secale Cornutum, with an Engraving. By T. H. WARDLEWORTH, Surgeon. Duodecimo, pp. 69. Simpkin and Co. 1840.

9. An Appeal to Parliament, the Medical Profession, and the Public, on the present State of Dental Surgery. By GEORGE WAITE, Surgeon-Dentist, &c. Pp. 29. Highley, Fleet-street. 1841.

10. On the Cure of Strabismus. By THOMAS ELLIOT, Surgeon, Carlisle. (From the Ed. Med. and Surg. Journal.)

11. The Cyclopædia of Anatomy and Physiology. Edited by ROBERT B. TODD, M.D. Part XXI. April 1, 1841.

12. Stammering, and other Imperfections of Speech, treated by Surgical Operations on the Throat. By JAMES YEARSLEY, M.R.C.S. Churchill, London, 1841.

13. Diseases of the Lungs, Part II. On Consumption. By HUME WEATHERHEAD, M.D. Price 2s. 6d. Highley, 1841.

14. Researches on Operative Surgery, &c. with Plates. By FLEETWOOD CHURCHILL, M.D. &c. Octavo, pp. 360. With numerous Plates. Dublin, Martin Keene and Son, 1841.

15. The Madras Quarterly Medical Journal. Edited by SAMUEL ROGERS, Assistant Surgeon Madras Establishment. Vol. II. for 1840.

16. Hints for Invalids about to visit Naples; being a Sketch of the Medical Topography of the City:—also an Account of the Mineral Waters of the Bay of Naples, with Analyses, &c. By J. C. COX, M.D. &c. Octavo, pp. 192. Longman and Co. 1841.

17. The Philosophy of Mystery. By WALTER COOPER DENDY, Fellow and Librarian of the Medical Society of London, &c. Octavo, pp. 443. Longman and Co. 1841.

18. Elements of Pathological Anatomy, illustrated by numerous Engravings. By S. D. GROSS, M.D. In two Volumes, 8vo. Boston, 1839.

☞ *Per favour of Dr. Caldwell.*

19. The Provincial Medical and Surgical Journal, No. 30, April 24th, 1841.

20. The Edinburgh Monthly Journal of Medical Science, No. 5. May, 1841.

21. A General Outline of the Animal Kingdom, and Manual of Comparative Anatomy. By THOMAS RYMER JONES, F.Z.S. &c. &c. Parts XIV. and XV. for February and May, 1841.

22. Hippopathology. Part II. The Diseases of the Digestive, Urinary, and Generative Organs of the Horse, with the different Methods of Castration. By WILLIAM PERCIVAL, M.R.C.S. &c. Veterinary Surgeon to the 1st Life Guards. London, Longman and Co. May, 1841.

23. On the Diseases and Derangements of the Nervous System, in their Primary Forms and in their Modifications, &c. &c. By MARSHALL HALL, M.D. &c. Octavo, pp. 380, with Plates. Bailliere. May, 1841.

24. Report of the Cases attended at the Birmingham Eye-Infirmary, during the Years 1838 and 9. By RICHARD MIDDLEMORE, Esq. Surgeon to the Infirmary. Octavo, pp. 20. 1841.

25. The Principles and Practice of Obstetric Medicine and Surgery, in reference to the Process of Parturition. With one hundred Illustrations on Steel and Wood. By FRANCIS RAMSBOTHAM, M.D., &c. &c. Obstetric Physician to the Eastern and Tower Hamlets Dispensaries. One Vol. octavo, pp. 672. Churchill, London, 1841.

☞ *In our next.*

26. Rambles in Europe, in 1839. With Sketches of prominent Surgeons, Physicians, Medical Schools, Hospitals, Literary Personages, Scenery, &c. By WILLIAM GIBSON, M.D. Professor of Surgery in the University of Pennsylvania, &c. Philadelphia, 1841. Churchill, London. May, 1841.

27. The Microscopic Journal, and Monthly Record of Facts in Microscopical Science, &c. Edited by DANIEL COOPER, M.R.C.S. &c. Part I. J. Van Voorst. May, 1841. Price 1s. 6d.

28. Transactions of the Provincial Medical and Surgical Association. Instituted 1832. Vol. IX. London, Churchill. May, 1841.

29. Annual Report of the Belfast District Asylum for Lunatic Poor. April 1840, to March 1841. Manager, Robert Stewart, M.D. resident.

30. The Philosophy of Death; or a General and Statistical Treatise on the Nature and Causes of Human Mortality. By JOHN REID, Licentiate of the Faculty of Physicians and Surgeons, Glasgow. Octavo, pp. 382. Glasgow and London, 1841.

31. The Anatomy of the Arteries of the Human Body; with its Application to Pathology and Operative Surgery, &c. in Lithographic Drawings, with practical Commentaries. Part VII. Price 12 shillings, including letter-press. Taylor and Walton, May, 1841.

32. The Sanative Influence of Climate; with an Account of the best Places of Resort for Invalids, in England, the South of Europe, &c. By Sir JAMES CLARK, Bart. M.D. F.R.S. &c. Third Edition. J. Murray. 1841.

☞ *This is nearly re-written, and greatly improved.*

33. Redstone's Guernsey Guide; or the Stranger's Companion for the Island of Guernsey, &c. Duodecimo, 1841.

---

34. On Gout: its Causes, Nature, and Treatment. By JOHN PARKIN, &c. &c. Octavo, pp. 140. Hatchard, Piccadilly. 1841.

---

35. A Treatise on Pyrosis Idiopatheca, or Water-brash, &c. By THOMAS WEST, M.D. Octavo. Longman's and Co. 1841.

---

36. Observations on Aneurism, its Surgical Pathology and Treatment, &c. By W. HENRY PORTER, A.M., Professor of Surgery in the Royal College of Surgeons in Ireland. Octavo, pp. 214. Dublin, 1841.

---

37. Report on the Mortality of Lunatics. By WILLIAM FARR, Esq.

---

38. Outlines of the Institutes of Medicine; including the Philosophy of the Human Economy in Health and in Disease. By JOSEPH A. GALLUP, M.D. Two Vols. 8vo. Boston, 1839.

---

39. A Winter in the Azores; and a Summer at the Baths of Furnas. By JOSEPH BULLAR, M.D. and HENRY BULLAR, of Lincoln's Inn. Two Vols. 8vo, with Lithographs. J. Van Voorst, 1841.

---

40. Traité des Neuralgies, ou Affections Douloureuses des Nerfs. Par F. L. VALLEIX, M.D. du Bureau Central de Paris, &c. One Vol. 8vo, pp. 718. Paris and London, Bailliere. 1841.

---

41. Odontography; or a Treatise on the Comparative Anatomy of the Teeth, their Physiological Relations, Mode of Development, and Microscopic Structure in the Vertebrate Animals. Illustrated by upwards of 150 Plates. By RICHARD OWEN, F.R.S. &c. Part Second, containing 50 Plates. Bailliere, London, May 1841.

---

42. A new Synopsis of the Natural Order of Diseases: containing their Definition, Principles, and Treatment, with a new Pathology of Fever and Inflammation. By ROBERT STEVENS, M.R.C.S. &c. Octavo, pp. 175. Highley. May 1841.

---

43. A Reply to Dr. Shirley Palmer's Letter, inserted in the Midland Herald. By Mr. GEORGE MILES MASON.

☞ *We regret these discussions on the differences between man and the monkey. The former must be distinguished from the latter by moral faculties rather than by anatomical structure.*

---

44. The History of British Birds. By W. YARRELL, F.L.S. &c. Parts 22, 23, 24, 25, illustrated by Woodcuts and numerous Vignettes. London, J. Van Voorst. 1841. Price 2s. 6d. each Part.

---

45. The Physiology of Vision. By WILLIAM MACKENZIE, M.D. Surgeon Oculist in Scotland to the Queen, &c. Octavo. Longman and Co. 1841.

---

46. On Stammering and Squinting; and on the Methods for their Removal. By EDWIN LEE, M.R.C.S. &c. Octavo, pp. 88. Churchill, London. 1841.

---

47. The Naturalist's Library, conducted by Sir WILLIAM JARDINE, Bart. Mammalia, Vol. XII. Horses. The Equido or Genus Equus of Authors. By Lieut.-Col. CHARLES HAMILTON SMITH, &c. Edinb. Lizars. London, Highley. June, 1841.

---

48. The Cyclopædia of Practical Surgery, &c. Edited by W. B. COSTELLO, M.D. Part VIII. May, 1841.

---

49. Some Account of the Scarlet Fever lately Epidemic in Liverpool. By J. R. W. VOSE, M.D.

---

50. Spinal Affections: a popular Lecture on Disorders of the Spine, &c. By HENRY CROWHURST ROODS, M.R.C.S. Duodec. Bailliere. May, 1841.

---

51. Phrenology consistent with Science and Revelation. By CHARLES COWAN, M.D. Physician to the Royal Berkshire Hospital, &c. Duodecimo, pp. 55. Sherwood and Co. London, June, 1841.

☞ *This is a very sensible little Treatise.*

---

52. An Inquiry concerning the Diseases and Functions of the Brain, the Spinal Cord, and the Nerves. By AMARIAH BRIGHAM, M.D. New York, 1840. Pp. 327.

---

53. Statements respecting Hospitals in China. By the Rev. PETER PARKER, M.D. Medical Missionary in China. Pp. 16. Suter, Fleet Street.

